# Prediction Models to Control Aging Time in Red Wine

**DOI:** 10.3390/molecules24050826

**Published:** 2019-02-26

**Authors:** Gonzalo Astray, Juan Carlos Mejuto, Víctor Martínez-Martínez, Ignacio Nevares, Maria Alamo-Sanza, Jesus Simal-Gandara

**Affiliations:** 1 Department of Physical Chemistry, Faculty of Food Science and Technology, University of Vigo, Ourense Campus, 32004 Ourense, Spain; gastray@uvigo.es (G.A.); xmejuto@uvigo.es (J.C.M.); 2 Department of Agricultural and Forestry Engineering, UVaMOX-University of Valladolid, Palencia Campus, 34001 Palencia, Spain; victor.martinez.martinez@uva.es (V.M.-M.); ignacio.nevares@uva.es (I.N.); 3 Department of Analytical Chemistry, UVaMOX-University of Valladolid, Palencia Campus, 34001 Palencia, Spain; 4 Nutrition and Bromatology Group, Department of Analytical and Food Chemistry, Faculty of Food Science and Technology, University of Vigo, Ourense Campus, 32004 Ourense, Spain

**Keywords:** food authenticity, toro appellation of origin, prediction models, wine, aging

## Abstract

A combination of physical-chemical analysis has been used to monitor the aging of red wines from D.O. Toro (Spain). The changes in the chemical composition of wines that occur over the aging time can be used to distinguish between wine samples collected after one, four, seven and ten months of aging. Different computational models were used to develop a good authenticity tool to certify wines. In this research, different models have been developed: Artificial Neural Network models (ANNs), Support Vector Machine (SVM) and Random Forest (RF) models. The results obtained for the ANN model developed with sigmoidal function in the output neuron and the RF model permit us to determine the aging time, with an average absolute percentage deviation below 1%, so it can be concluded that these two models have demonstrated their capacity to predict the age of wine.

## 1. Introduction

In the last decade, consumers have become interested in foods identified with a place of origin [1], and in their characteristics and quality [1,2]. One of these products is wine, a beverage obtained from the alcoholic fermentation of grapes [3]; it is one of the most popular [4], complex [5] and consumed alcoholic beverages around the world [6]. In the European Union (EU), wines produced in specified regions are clearly identified and controlled [7]. In this sense, there are different quality schemes under a geographical indication according to specific characteristics: (i) protected designation of origin (PDO), (ii) protected geographical indication (PGI) and (iii) geographical indication of spirit drinks and aromatized wines (GI) [8]. As is understandable, the use of these schemes impacts on market recognition and even results in higher sale prices. For this reason, the improper use of these geographical indications can be injurious to producers and consumers [1]. South European countries (Spain among others) are involved in food authentication studies, for example in wines and foodstuffs registered as, among others, PDO [9].

Wine adulterations such as water dilution or mixing with cheaper wine are common [10]. Nowadays, quality and commercial value are linked to elaboration procedures and geographical places [10], as for example, Tempranillo red wine from D.O (*Denominación de Origen*) Toro (Spain), where the wine authenticity is a key factor in terms of differentiation, which has a significant influence on the final sale price [10].

A wine’s quality and organoleptic properties can be influenced by oenological parameters such as grape variety and winemaking process, among others [11]. Its quality can be evaluated through chemical/biological tests and sensory tests using different classification models [12]. Due to these parameters being related to the wine´s quality/price, the possible to find a relationship between physicochemical parameters and the wine’s age (D.O. Toro) can be interesting, especially if the wine´s characterization and its combination with chemometric treatment can provide good results that also reduce the operative costs compared to other methods like the use of expert panellists [2].

In the literature, different methods for wine age prediction have been reported, such as single block regression methods [13], multiblock regression methods [14] and latent variable methods [15,16,17]. In this research, four different models are presented: (i) two Artificial Neural Networks models (ANNs), (ii) one Support Vector Machine (SVM) model, and iii) one Random Forest (RF) model.

### Related Works WITH This Research

ANNs are a computational technique which draw upon biological neural system [18,19,20,21]. In their research, McCulloch and Pitts [22] introduced the concept of the artificial neuron [23]. These interconnected units (artificial neurons or nodes) are able to model complex nonlinear relationships between independent variables (also called inputs) and dependent variables (outputs) [20,24]. ANN models based on a multi-layer perceptron (MLP), one of the most popular ANN topologies [23], were used in this research. An MLP is a feed-forward ANN model that maps input data onto output data [25]. This kind of model has multiple layers of neurons (input, hidden and output), with each layer being connected to the next network layer [25]. One of the most important advantages is that ANN models can extract information from complex data matrices due their ability to learn the relationship between independent and dependent variables [26]. For this reason, ANNs are applied in many different research fields, such as:

(i) Hydrology, to model the water quality using different water quality variables [27],
(ii) Biotechnology, to optimize 1,3-propanediol production using microorganisms like *Lactobacillus brevis* N1E9.3.3 [28] or to optimize oil extraction from *Bauhinia monandra* seed [18],
(iii) Food technology, to develop an authentication model to predict the cultivar, the production type and the harvest date for tomatoes [29] or to authenticate extra virgin oil varieties [30],
(iv) Chemistry, to predict percolation temperature [31], to predict the solvent accessibility of proteins [32], or in other fields where the ANN has proved its capacity for medical, economic or agro-food science purposes [21].

Support vector machines were first introduced by Boser et al. in 1992 [33,34]. The SVM is a powerful non-linear method to develop classification and regression models [25,35]. A SVM model uses input data to construct a hyperplane, or a group of hyperplanes, in a high-dimensional space [25]. These hyperplanes allow the SVM model to be used for different purposes [25]. Its main advantage, in comparison with other classification techniques, for example, Partial Least Squares-Discriminant Analysis (PLS-DA), is that it is flexible to model complex classification non-linear problems [35]. For this reason, Support Vector Machine models can serve in many studies and applications such as:

(i) To determine air specific heat ratios at elevated pressures [36],
(ii) to classify glaucoma, a progressive optic neuropathy disease [37],
(iii) to forecast electrical loads due to their importance in the regional power system strategy management [38] or,
(iv) to evaluate real-time crash risk in active traffic management (ATM) [39], among other fields.

Random forest is a learning method for classification or regression [40,41] that was proposed by Breiman in 2001 [41,42]. RF models consist of a classifier with different decision trees, where the final prediction is obtained by all the single classification trees [41,43], that is, for a quantitative response, the prediction is the average of each individual tree predicted value [44]. This ability is the key that converts Random Forest into a powerful prediction method [44]. Random forest corrects the problem of overfitting that presents the decision trees [40], and has been used in multiple research fields, such as:

(i) Medicine, to estimate the survivability of cancer patients [40],
(ii) Food technology, to develop a model focused on the volatile organic compounds responsible for the olfactory perception [44],
(iii) Ecology, to classify invasive plants [45], or to estimate high-density biomass for wetland vegetation [46], inter alia.

Therefore, the main objective of this paper is to develop different prediction models as tools for determining wine authenticity, i.e., that could predict the aging time (1-4-7-10 months) of red wines from D.O. Toro (Spain).

## 2. Results and Discussion

The use of alternative products (chips, staves...) is a common practice in the winemaking sector. However, the differentiation of wines according to their aging system is very complicated. During the ageing process of the same red wine with alternative products+MOX, a series of process reactions take place as a result of the wine coming into contact with the wood [47]. The toasting level of the oak wood is very important, as it regulates the transfer of the compounds from the wood to the wine. During wine ageing, the higher the toasting level of the oak wood, the more substances responsible for the spicy and smoky aromas can be appreciated [48]. Aging with chips is faster than traditional barrel aging, so wines have a more volatile acidity and more phenolic compounds than barrel wines. However, the differentiation of wines is complicated, and work is needed on the development of tools for differentiation. Numerous ANN models were developed using a trial and error method to find the best neural model topology. Over seven thousand neural network models with different topologies and training cycles were developed (varying the number of intermediate neurons between one and 2*n*+1, where *n* is the number of input variables). The best neural model was chosen based on its validation performance, and then the best models were rechecked with the querying data group.

Table 1 shows the adjustments for the best ANN_1_ model selected. It can be observed that the neural model developed with linear function in the output layer presents a good determination coefficient in all phases (between 0.998 for the validation phase and 0.989 for querying phase). For the training phase, the error is below 10% (an acceptable error for this type of variable -aging time-). Similar behaviour is observed in the validation phase. In both phases, the root mean squared error in under 0.29 months. In the querying phase, the ANN_1_ model presents a good R^2^ (0.989); nevertheless, a slight worsening is observed in the prediction in terms of root mean squared error (RMSE = 0.40 months) and average absolute percentage deviation (AAPD = 13.51%).

**Figure 1:**
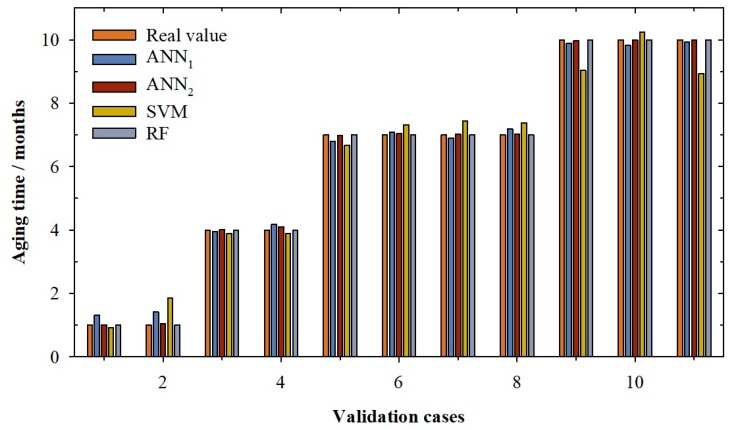
Bar graph for validation cases according to the real value of aging time (light brown) and the values predicted by the artificial neural network with linear function in output neuron (ANN_1_, dark blue), artificial neural network with sigmoidal function in output neuron (ANN_2_, dark brown), support vector machine (SVM, olive) and random forest (RF, light blue).

**Figure 2:**
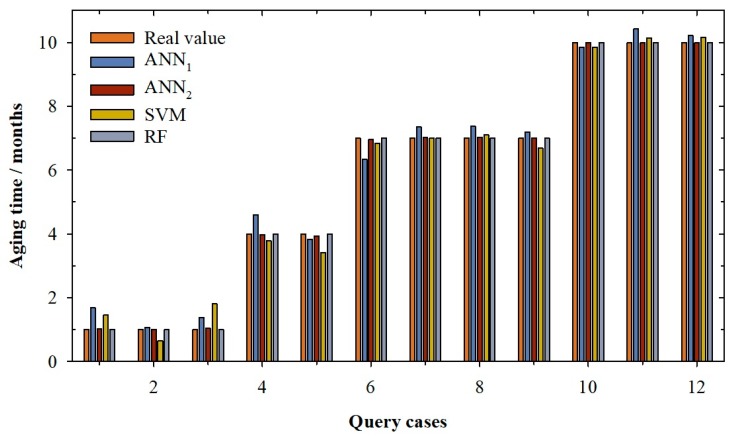
Bar graph for querying cases according to the real value of aging time (light brown) and the values predicted by the artificial neural network with linear function in output neuron (ANN_1_, dark blue), artificial neural network with sigmoidal function in output neuron (ANN_2_, dark brown), support vector machine (SVM, olive) and random forest (RF, light blue).

Figure 1 and Figure 2 show the real value of aging time (light brown) and the values predicted by the best ANN_1_ model (dark blue) developed in this research. It can be observed in the validation cases (Figure 1) that the ANN_1_ model overestimated the real value for the cases 1 and 2, while for cases 4, 6 and 8, the overestimation is very slight (between 1.28% and 4.46%). Cases 1 and 2 present a high error; in fact, the real value is 1 and the values predicted were 1.31 and 1.41, respectively. For the rest of the validation cases, the estimates are slightly lower than the real values (between -0.81% and -2.84%). For query cases (Figure 2), it can be seen how the linear ANN model presents overestimation of the aging time value in nine of the twelve cases reserved (especially in the cases 1, 3 and 4). Once again, the cases with real aging time of 1 month were those with bigger errors. Cases 1 and 3 present an individual percentage deviation of 68.70% and 37.25%. This is the reason for the increase of RMSE and the AAPD values in the querying phase. This behaviour is also observed in the training phase, where cases with one month of aging show greater errors between -0.91% and 69.43% (1 month vs. 1.69 months predicted). This is probably because there is still no significant differentiation between wines, since they have only been in contact with wood for one month, and it is possible that in each system the speed of aging is different. In view of these results, it can be concluded that the ANN_1_ model presents a general good performance in all its phases, but that for low aging times, the model does not work at all well. 

The next model developed was the ANN model (ANN_2_) with logistic function in its output neuron. As can be seen in Table 1, the adjustment parameters improve the fits of the ANN_1_ model. It can be seen that for the training and the validation phase, the model presents coefficients of determination of one, improving the R^2^ of the ANN_1_ model. It is also clear that the ANN_2_ model improves the adjustments in terms of RMSE and AAPD, going from an RMSE of 0.20 months to 0.04 months, for the validation phase of the ANN_1_ and ANN_2_ model, respectively. This good behaviour is also observed in the querying phase where the ANN_2_ model presents a good determination coefficient (R^2^ = 1.000), which corresponds with a low value of root mean squared error (RMSE = 0.03 months) and an average absolute percentage deviation below 0.85%.

In Figure 1, the real value of aging time (light brown) and the values predicted by ANN_2_ model (dark brown) for validation cases can be seen. The ANN_2_ model predicts with accuracy the real value of aging time. This behaviour is also observed for the query cases (Figure 2); it can be seen how the logistic ANN model presents a good prediction of the aging time value for all cases which means that the adjustments of this phase are good (0.03 months of RMSE and 0.84% of APPD. In contrast to the previous ANN model, in this model, high errors were not observed in any of the aging periods studied; in fact, individual percentage deviation remains between −1.63% and 3.99%. With these results, it can be said that ANN_2_ can predict with accuracy the aging time of red wines from D.O. Toro (Spain). 

A new model based on a support vector machine was developed using library LIBSVM by Chang and Lin [25,49]. Gamma and C values were studied using a trial and error method to find the best combination according to the range proposed by the updated guide provided by Hsu et al. (2003) [50].

In Table 1, the adjustments for the selected SVM model can be seen. It can be observed that the model presents a good determination coefficient in the training phase (0.995) with a low APPD, i.e., around 6.72% (RMSE of 0.24 months). For the validation phase, it can be seen how the value of the determination coefficient falls slightly to 0.973 and the average absolute percentage deviation increases to 12.86%, corresponding with a root mean squared error of 0.56 months. This high AAPD in the validation phase is due to the case number 2, in which the model predicts an aging value of 1.85, when the real value is 1 month, that is, the model predicts this case with an 85.12% of individual percentage deviation (see Figure 1). This high error significantly affects the model’s AAPD value for the validation phase (12.86%, see Table 1). Another two cases, 9 and 11, present an error close to the one considered as acceptable (10%), −9.64 % and −10.73%, respectively. The same behaviour can be seen in the querying phase. In this case, the R^2^ increases to 0.988 and the RMSE decreases to 0.37 months. Nevertheless, the APPD increases to 16.35%; this value is, once again, due to the prediction for cases with one month of aging time (see Figure 2). For cases 1 and 3, the individual percentages deviations are around 46.07% and an 80.85%, respectively, while the individual percentage deviation for case 2 is −36.03%. These high values distort the value of the APPD in the querying phase. In view of these results, it can be concluded that the SVM model presents bad results for low aging times.

Finally, the last model developed in this research is a Random Forest model. According to the parameters exposed above, the best Random Forest model is the one with only one tree, which provided the results shown in Table 1. It can be observed that the model presents an optimum determination coefficient which causes the other analysed parameters, RMSE and APPD, to be zero (Table 1). The random forest model found that the variables that dominate the determination of aging time are the total-SO_2_ (T-SO_2_), the alcoholic grade (AD) and the free-SO_2_ (F-SO_2_). During wine aging, both free SO_2_ and combined SO_2_ is lost over time. These losses are due to the interactions of SO2 with wine components and with the oxygen that the wine receives during the process. In addition, with aging time, the wine suffers a slight concentration effect due to the evaporation processes that occurs in the barrel, increasing its alcoholic degree. A random forest with only one tree and with these three parameters is enough to predict with total accuracy all cases of the training, validation and querying phase (Figure 1 and Figure 2). These results show that the RF model can predict the aging time with accuracy.

It seems clear that the adjustments obtained for the ANN_1_ and SVM models are not good when wines with only one month of aging come into play. For other aging times, both models work reasonably well. The results obtained for ANN_2_ (developed with thirteen input variables) and the RF model (that used three input variables) can be used to guarantee red wine aging authenticity. These two models are able to predict, with accuracy, the aging time with, in the worst case scenario (ANN_2_), an average absolute percentage deviation below 1%, which corresponds to a maximum error of 0.04 months (in terms of RMSE). These results improve the principal component analysis (PCA) model developed by Apetrei et al. (2012) using the oenological parameters where the analysis can describe a 61% (28% for the first principal component, of the information; 21% for the second and a 12% for the third) [51]. The partial least squares-discriminant analysis (PLS-DA) using the physicochemical analyses can only explain 59% of the variance in calibration and 77% in predictions presenting an RMSE up to 0.347 [51].

Regarding the RF model, and to our understanding, a single tree in the random forest model seems to indicate that the wines of the Toro designation of origin studied in this research show particular characteristics that can be a key factor in predicting aging times. In addition to this, it is expected that the inclusion of new experimental data from different wines, batches and months could lead to the development of RF models with more trees.

## 3. Materials and Methods

### 3.1. Data Set

A red wine, variety Tempranillo or Tinta de Toro belong to D.O. Toro (Spain), was studied. A total of 16 batches were used, which had undergone 3 different aging systems, traditional (barrels), alternative (chips+microxygenation, MOX) and stainless steel tank. Nine 210 L stainless steel tanks were used to study alternative wine aging: 1-2-3) chips with light toast level and MOX; 4-5-6) chips with medium toast level and MOX; 7-8-9) chips with heavy toast level and MOX. The traditional aging system was studied in 6 barrels of light, medium and strong toast level in a duplicated way. Both the barrels and the chips were made with oak French (allier, Q. *sessilis*) by the same cooperage (Doreau, France). All tanks were used with small doses of oxygen (MOX) with an equipment (OenoAZ3) simulating the micro-oxygenation which occurs due to the wood pores in the barrel, the microoxygenation in the range 2.0–3.0 mL L^−1^ month^−1^ O_2_. In addition, a control (without contact with wood) wine in a stainless steel tank was studied during the experiment. In this research, the 58 samples reported by Apetrei et al. (2012) in their original research were used [51].

### 3.2. Physical-chemical Analysis

Independent variables were obtained by Apetrei et al. (2012) using conventional chemical analyses of the wines according to international regulations of International Organisation of Vine and Wine [52]. These parameters were: tartaric acid (T) using a colorimetric method, glycerol (G) using a gases chromatography method, potassium (K) using an atomic absorption spectroscopy method, total polyphenol index (TPI) using a spectrophotometric method, total acidity (TA) using a potentiometric method, alcoholic grade (AD) using a distillation method, dry extract (DE) using densimetric method, volatile acidity (VA) expressed as acetic acid using an enzymatic method, total-SO_2_ (T-SO_2_) and free-SO2 (F-SO2) according to an iodometric method, reducing sugars (S) using an enzymatic method, relative density (DEN)according to electronic densimeter method and pH. All methods are certified by the National Accreditation Entity (ENAC).

### 3.3. Methodologies

According to the main purpose of this research, it is possible to locate in the literature artificial neural networks, support vector machines and random forest models focused on different fields related to wine. It is possible to find research papers about neural models conceived to verify the origin of wines [53], to classify Slovak white wines from different producers, varieties and production year [54], or for geographical classification [3,55], among others. Additionally, SVM has been used to classify Syrah wines according to their origin (Mendoza—Argentina- and Central Valley—Chile-) and then compared with neural networks [3] to authenticate wines from South Africa, Hungary, Romania and Czech Republic [33], to characterize and authenticate different Spanish PDO wine vinegars (Vinagre de Jerez, Vinagre de Montilla-Moriles and Vinagre del Condado de Huelva) [35], to predict enological parameters and determine rice wine age [56] or to predict wine’s grade [4], inter alia. Finally, Random Forests have been used to classify wines according to their production regions using trace elements [41], to model the impact of climate change on wine regions (Hungary) [57] or in different European wine regions [58], and to classify the cultivars on the basis of different chemical present in wine [59], among others.

The first model developed was an ANN model. To obtain the best ANN model, it is necessary to develop different ANN topologies with many configuration options using a trial and error procedure [23,60]. The ANN model’s topology is composed of different kinds of layers: (i) a first layer (called input layer) is destined to introduce the experimental data into the network, (ii) next, another kind of layer or layers (called hidden or intermediate), and finally, (iii) a last layer (output layer) where the predicted value is generated. During the ANN training phase, the value connection between neurons (called weights) is adjusted to achieve the minimum error between the experimental and the predicted output [61]. This process occurs in the hidden layers and output layer, and allows the neural network to learn based on training experimental cases. A trial and error approach was used to find the best neural model. Different topologies and training cycles were used to provide the best results according to statistics in the validation phase.

In this research, two types of ANN have been analysed. The first network, ANN_1_, with a backpropagation algorithm, sigmoidal function in its intermediate neurons and a linear function in the output neuron, and a second type, ANN_2_, also with backpropagation algorithm and sigmoidal function in all intermediate and output neurons. A disadvantage of neural models based on the back-propagation algorithm is that it requires huge computational time to optimize the different parameters which constitute the neural model [3,62]. For this reason, other techniques such as SVM and RF have been studied to reduce computational costs and times of execution. SVM is a powerful technique for classification and regression [25]; in our case, it was used for regression tasks using epsilon-SVR and nu-SVR SVM types [25]. The SVM model finds an optimum separating hyperplane to maximize the borderline of the decision surface [3]. In this study, the LIBSVM learner by Chang and Lin [25,49] was used. Our SVM model used the RBF kernel; the configuration of parameters, gamma and C, were studied according to the range proposed by the updated guide provide by Hsu et al. (2003) [50].

In the Random Forest regression model, three parameters were optimized: (i) the number of trees (1 to 100 in twenty linear steps), (ii) the least square criterion, (iii) maximal depth (−1 to 10 in eleven linear steps), and (iv) apply pre-pruning (true or false).

### 3.4. Model’s Prediction Statistics

Data from the original paper were split randomly into three groups, one group used to develop the model (called training group, 35 cases), another formed by 11 cases (validation group) used to validate the model, and a third group to query the selected model (querying group, 12 cases). In this research, the predictive power of the different models was determined as a function of the coefficient of determination (R^2^), the Root Mean Squared Error (RMSE) (Equation (1)) and the Average Absolute Percentage Deviation (AAPD) (Equation (2)).

(1) $$\mathrm{RMSE}=\sqrt{\frac{\sum_{t=1}^{n}{\left({\hat{y}}_{t}-y_{t}\right)}^{2}}{n}}$$

(2) $$\mathrm{AAPD}=\frac{100\%}{n}\overset{n}{\underset{t=1}{\sum }}\left|\frac{{\hat{y}}_{t}-y_{t}}{y_{t}}\right|$$

### 3.5. Equipment and Software

Neural models have been implemented in an AMD Ryzen 7 1800X Eight-Core Processor 3.60 GHz (Advanced Micro Devices, Inc, Sunnyvale, CA, USA) with 16 GB of RAM memory. ANN_1_, SVM and RF models were developed using a Trial/Free version of RapidMiner Studio from RapidMiner Inc (Boston, MA, USA). The Neural model ANN_2_ was developed using the EasyNN plus v14.0d software from Neural Planner Software Ltd (Cheadle Hulme, England). Data were fitted using Microsoft Excel from Microsoft Office Professional Plus 2013. Figures were drawn with Sigmaplot 13 from Systat Software Inc (San José, CA, USA).

## 4. Conclusions

In this study, different models were developed to monitor red wines from D.O. Toro (Spain). In view of the results obtained by the models, ANN_1_ and SVM, it would be advisable to continue with the analysis of wines of the D.O. Toro, and even to incorporate wines from close appellations of origin. The results obtained by the ANN model with sigmoidal function in the output neuron and the random forest model, which used physical-chemical parameters, allowed us to determine aging times, with an average absolute percentage deviation of below 1%.

## Figures and Tables

**Table 1:** Coefficient of determination (R^2^), root mean squared error (RMSE) and average absolute percentage deviation (AAPD) for training, validation and querying phase, for each model present in this research (artificial neural network with linear function in output neuron (ANN_1_), artificial neural network with sigmoidal function in output neuron (ANN_2_), support vector machine (SVM) and random forest (RF)).

	Training			Validation			Querying		
Model	R^2^	RMSE	AAPD (%)	R^2^	RMSE	AAPD (%)	R^2^	RMSE	AAPD (%)
**ANN_1_**	0.994	0.28	8.07	0.998	0.20	8.20	0.989	0.40	13.51
**ANN_2_**	1.000	0.02	0.42	1.000	0.04	0.87	1.000	0.03	0.84
**SVM**	0.995	0.24	6.72	0.973	0.56	12.86	0.988	0.37	16.35
**RF**	1.000	0.00	0.00	1.000	0.00	0.00	1.000	0.00	0.00
